# The Impacts of Car-Free Days and Events on the Environment and Human Health

**DOI:** 10.1007/s40572-022-00342-y

**Published:** 2022-02-10

**Authors:** Andrew Glazener, James Wylie, Willem van Waas, Haneen Khreis

**Affiliations:** 1 Community and Regional Planning Program, School of Architecture, University of Texas at Austin, Austin, TX USA; 2 Solar Impulse Foundation, Lausanne, Switzerland; 3 Department of Traffic and Public Space, Municipality of Amsterdam, Amsterdam, The Netherlands; 4 MRC Epidemiology Unit, Institute of Metabolic Science, University of Cambridge School of Clinical Medicine, Cambridge Biomedical Campus, Cambridge, CB2 0SL UK; 5 Center for Advancing Research in Transportation Emissions, Energy, and Health (CARTEEH), Texas A&M Transportation Institute (TTI), College Station, TX 77843-3135 USA

**Keywords:** Car-free, transportation, cities, public health, air pollution, physical activity

## Abstract

**Purpose of review:**

In this paper, we seek to elucidate the impact of car-free days and events on human health. Car-free days and events are often designed to alleviate the impact of transportation-related air pollution, noise, physical inactivity, traffic congestion, or other detrimental externalities of private motor vehicle travel. We reviewed existing peer-reviewed and gray literature to understand the variety of potential public health impacts that have been measured as a result of car-free days or events and associated changes in environmental exposures and lifestyles.

**Recent findings:**

The impacts of car-free days and events are highly variable and seem to depend on the scope (frequency, duration, and geographic size) and goals of each car-free day and event. Most of the existing literature measures impacts in terms of air and noise pollution and some studies focus on physical activity metrics. In some cases, car-free days and events were successful in reducing the concentration of certain air pollutants but had little or adverse impacts on the concentration of others. Often, traffic is diverted from cordoned areas to surrounding streets, displacing traffic congestion and adverse environmental exposures to other areas of a city, with potential understudied implications to environmental justice.

**Summary:**

Car-free days and events are often an attractive policy option; however, they require intensive planning to be successful. The organization and execution of car-free days and events, as well as public support and stakeholder engagement, greatly influence the level of success and the sustainability of such initiatives. Health benefits may be a palatable and convincing argument to the general public. However, very few studies focus on actual health impacts associated with car-free days and events. Future research could be most useful if it focused on measuring health outcomes associated with car-free days and events through longitudinal studies.

## Introduction

The advent of automobility unlocked dimensions of transportation previously unavailable—a degree of access, mobility, freedom from scheduled and fixed route systems, and speed that “subordinated” other available modes [1, 2]. There are an estimated 2.2 billion registered motor vehicles (including motorcycles) globally, a number that is expected to increase in the years to come and double by 2040 [3]. China (290 million), the USA (280 million), and India (210 million) account for the highest number of registered vehicles in the world [3]. Developing nations—particularly India and China—are expected to see the largest increase in car ownership over the coming decades compared to other nations [4]. In 2019, however, global motor vehicle sales totaled 51.4 million, down from 55 million sold in 2018 and the lowest since 2011 [5].

Cars became ingrained in modern society throughout the twentieth century for multiple reasons. First, the Model T car produced by the Ford Motor Company was one of the early products to optimize the benefits of industrialization, technological and design innovations, and mass production, which allowed Ford to reduce manufacturing costs and provide the first widely affordable mode of motorized transportation (The Model T) [6].

Second, the automotive industry provided an important source of jobs, peaking in the USA in the early twenty-first century [7]. This success exemplified the economic potential of the automotive industry and countries such as France, Germany, Italy, Japan, and South Korea also expanded their economies through the automotive industry [8]. Despite early successes, the adverse impacts of the automotive industry are exemplified through the history of Detroit, USA, which underwent a historic population increase provoked by the economic ascent of automotive manufacturing in the early twentieth century. This was followed by decades of decline as factories (and jobs) vacated the city and devastated the local economy and was apparent in other states of the American Midwest [7, 9]. Similar processes of decline and automotive de-industrialization have occurred in regions of Canada and the UK [10, 11]. Nearly 60% of car manufacturing in the U.K. was located in the West Midlands at the beginning of the 1970s, however, only 18% of car manufacturing still occurred in the region in 2008, corresponding with a 70-80% reduction in automotive jobs among certain manufacturers in the region [10]. More recently, Oshawa, Canada was the wealthiest Canadian city for a decade due to large automotive manufacturing plants until a decline in the automotive industry eliminated over one-third of automotive jobs and diminished the economic well-being of the population as a whole [11].

Third, as car ownership proliferated, cars have become central to the way we build cities and use land, interact with the built environment, satisfy our travel needs, and much more [12]. The convenience as represented in reduced travel time, increased comfort, improved accessibility, and ease of mobility compared to other modes in the early twentieth century made them attractive options. The dependence on cars manifested itself in the urban built environment in the form of low-density sprawl, single mixed-use developments, extensive road networks, and enormous parking lots. In many cities, zoning regulations mandate a minimum provision of parking spaces to improve automobile access to amenities. Parking minimums, however, often overestimate the necessary volume of parking spaces which results in higher development costs due to the high cost of parking construction [13]. The growth of cities and changing spatial distribution of populations as transportation modes evolved from walking, to streetcars, to automobiles clearly express the impact of the car on urban form [14]. Further, the invasion of cars into public space due to car-oriented development has revealed the spatial inefficiency of cars and their adverse impact on urban lifestyles, raising questions about the “fairness” of how road space, and therefore urban space, is allocated [15–17]. An “ego-enhancing medium” [18], cars also became symbols of status and privilege, further deepening the significance of car ownership in urban societies [2].

Economic assessments have been used in the past to estimate the relative cost of various transportation modes. Cars are estimated to produce external costs that society at-large must bear, most notably: vehicle crashes, air pollution, time lost due to congestion, physical inactivity, and urban space consumption [19]. Litman (2009) estimated the greatest cost reduction for the public could be achieved if car trips were replaced with walking and cycling, largely due to the health benefits of active travel [19]. These findings are supported by other cost–benefit analyses that suggest car travel incurs a fiscal burden on societies whereas active travel modes produce cost-savings mainly through the health benefits of physical activity [20, 21].

Recent work has identified 14 pathways that link transportation and health, 11 of which are associated with negative health outcomes [22••]. These 11 pathways are: physical inactivity, air pollution, motor vehicle crashes, noise, heat, stress, community severance, social exclusion, greenhouse gases, contamination, and electromagnetic fields. Dependence on cars, and its impact on the built environment exacerbates the negative health outcomes associated with these 11 pathways. Three pathways—access, green space, and mobility independence—were identified as producing positive health outcomes. The adoption of car-free initiatives can reduce the health burden of cars (such as decreasing air pollution, road-traffic noise, and motor vehicle crashes) and expand opportunities for health-promoting transportation practices by encouraging non-motorized transportation modes and physical activity.

In 2019, an estimated 90% of the global urban population lived in areas where average fine particulate matter (PM_2.5_) concentrations exceeded the World Health Organization (WHO) air quality guidelines [23]. Transportation-related air pollution (TRAP) can account for up to 53% of PM_10_ and 66% of PM_2.5_ concentrations in European cities, although the prevalence of TRAP depends on location-specific factors and some cities may not be characterized by high TRAP concentrations [24]. The countries with the highest average annual transportation-related PM_2.5_ concentrations are located in Southeast Asia (where vehicle ownership is also anticipated to increase the most [4]), Southwestern Europe, and India [25]. Transportation-related PM_2.5_ was estimated to cause 107,000 annual premature deaths in the USA [26] and 137,000 annual premature deaths in China [27]. Anenberg et al. (2019) showed that transportation-related emissions increased levels of ozone and PM_2.5_ which were estimated to account for 385,000 premature deaths globally in 2015 [28].

Motor vehicle crashes are the leading cause of death globally among 5-29 years old and are responsible for 1.35 million premature deaths each year across the total population [29]. The total burden of disease associated with transportation noise in Europe (including rail, road, and air traffic) is estimated to be comparable to that of second-hand smoke [30]. Many cities around the world are characterized by inhospitable levels of traffic congestion—the average car commuter in the 10 most congested cities will roughly spend 162 hours in traffic annually [31], which is found to reduce social ties and opportunities for social interaction [32]. The adoption of cars as the preferred travel mode has increased the amount of sedentary behavior individuals engage in [33]. Traveling by car is the least active mode, followed in ascending order by public transportation, walking, and cycling [34]. Car-oriented lifestyles can partly explain the growth of physical inactivity as one of the leading causes of premature death globally (5.3 million deaths in 2008). Cumulatively, physical inactivity, motor vehicle crashes, and TRAP account for over 7 million premature deaths globally, and this burden is only a snapshot of the real health burden as it does not account for other important risk factors such as traffic noise, contamination, stress and the health impacts of climate change.

The contribution of cars and road transportation to climate change is perhaps the gravest health risk. Greenhouse gases (GHGs) trap heat from solar radiation within the Earth’s atmosphere which contributes to the warming of Earth’s climate as more GHGs are emitted. Carbon dioxide (CO_2_) is the most common GHG, composing 76% of global GHG emissions [35]. In the USA, transportation emits more CO_2_ (28%) than any other sector [36]. Globally, transportation is responsible for 23% of all CO_2_ emissions [35]. The Intergovernmental Panel on Climate Change estimates that global GHG emissions need to be reduced by 45% of 2010 volumes by 2030—before ultimately achieving carbon neutrality by 2050—to avoid a global warming of 1.5°C [36]. Global warming is directly harmful to health through increased risk of heatwaves, droughts, natural disasters such as wildfires, floods, and storms, higher levels of ambient air pollution, adverse impacts on food systems and infectious disease vectors, and potential ecosystem collapse [37, 38]. Reducing the use of cars would limit urban GHG emissions, improve existing urban environmental conditions, and result in healthier behaviors among urban dwellers [39].

While directly limiting automobility would technically be the most desirable option, this meets a lot of resistance including from policy makers, car lobbies, and the population [40, 41]. In this light, so-called transition experiments are useful to let policy makers and urbanites experience the benefits of reductions in, or bans of, automobility in certain areas. Car-free days or events are planned efforts to temporarily limit or restrict private car use and prioritize movement by walking, cycling, and public transportation instead. Early on, car-free Sundays were held to limit oil consumption, such as in the Netherlands in 1939 and 1956 and in several countries during the oil crisis in 1973-4 [42]. Since the beginning, automobility has been controversial [43], but especially since the 1970s, resistance against the negative effects of automobility has become more pronounced (such as the Dutch “stop the child murder” movement) [44]. Initial adoption for car-free initiatives can also be traced to cities such as Bogotá, Colombia, Venice, Italy, and Zurich, Switzerland as early as the 1970s, eventually becoming more widely adopted in Europe during the 1990s and early 2000s [45]. The movement to encourage non-motorized, active travel could be a useful strategy to reduce dependence on cars and produce environmental and public health benefits.

The scale of car-free days or events is highly variable. Some car-free activities occur once, others annually (World Car-free Day), while still others recur as frequently as once per week, and are strictly enforced and supported by local governments, with defined geographic bounds within cities (e.g., *Ciclovía* in Bogotá, Colombia). The rationale for car-free days or events vary from addressing environmental and climate change concerns [46], pursuing transportation equity [47], and visioning how alternative mobility can improve general quality of life (health, social cohesion, etc.) [48]. The general underlying motivation of car-free days and events, however, is to prioritize alternative modes of transportation to the car.

Recently, stay-at-home and work-from-home orders prompted by the COVID-19 pandemic drastically reduced motor vehicle traffic and increased the demand for outdoor recreational space [49–52]. In response, some cities began closing streets off to cars to create more pedestrian and cyclist-friendly street environments that facilitate socially distanced recreation and exercise [53]. The benefits of these temporary car-free initiatives have encouraged citizens to demand these spaces remain car-free as restrictions are lifted [54].

The goal of this paper is to review examples of car-free days and events from around the world. The review includes academic and gray literature that discuss the emergence of car-free days and events, and their public health implications. Car-free initiatives have become increasingly popular and this paper synthesizes key examples of such initiatives and distills their health impacts. We also briefly discuss barriers and facilitators to the implementation of car-free initiatives. While the review of health impacts pertains to car-free days and events, the discussion of barriers and facilitators is broadened to include car-free city initiatives because the body of literature discussing barriers and facilitators largely focuses on car-free cities, not days and events.

## Literature Review

Given the limited nature of the literature related to the health effects of car-free days and events, maximum retrieval was strived for in this review. A combination of scientific and gray literature was consulted. The gray literature should be treated with caution, as documented effects may be confounded by for example weather conditions or other changes in the larger environment, which journalists do not always account for. For this reason, our conclusions will be primarily based on the peer-reviewed scientific literature. A limited range of literature on the barriers and facilitators specifically for car-free days and events was found, and as a result, a wider review was conducted to include literature on facilitators and barriers of other car-free initiatives (e.g., pedestrianization schemes), despite the differences in scope and style between partial and permanent car-free programs. We decided to include this wider body of literature to complement the limited information on barriers and facilitators, as we think that some of the factors that may deter or facilitate permeant bans and singular days and events are similar; for example, altering public acceptability and engaging different stakeholders such as affected businesses.

A range of search terms and databases was used, including backward and forward snowballing (i.e., papers in the reference list and papers that reference the article, respectively) to maximize inclusion. This way, four additional articles were obtained. For gray literature, searches in Google were conducted. For scientific literature, Scopus and Google Scholar were used. Searches were conducted by AG and WvW. A list of search terms is provided in Table 1.

**Table 1:** Literature Review Search Terms

Database	Search terms
Scopus	“car free” OR “traffic diversion” AND “health” OR “air” OR “safety” AND “day” OR “experiment” OR “festival”
	“Ciclovia” “car-free” “barrier”
	“car-free” “day” “ciclovia”
	“Car-free” AND “opposition” AND “day”
	“car-free day” OR “car-free event” OR “car free day” OR “car free event” OR “carfree days” OR “carfree events” AND “health”
	“green Sundays” OR “ecological Sundays” AND “health”
	“united states” AND “street closure” OR “limited car access” AND “health”
Google	car free days and events impact on public health
Google Scholar	“car-free day” OR “car-free event” OR “car free day” OR “car free event” OR “carfree days” OR “carfree events” AND “health”
	“green Sundays” OR “ecological Sundays” AND “health”
	“car-free day” OR “car-free event” OR “car free day” OR “car free event” AND “social” OR “community” AND “health”
	“car-free day” OR “car-free event” OR “car free day” OR “car free event” AND “congestion” OR “traffic” OR “delay”
	“car-free day” OR “car-free event” OR “car free day” OR “car free event” OR “carfree days” OR “carfree events” AND “noise” OR “physical activity” OR “greenhouse gas”

Importantly, only the effects of car-free days and events were included. The effect of traffic reductions on (among others) pollutant concentrations can be studied based on other phenomena such as Sundays or holidays but these studies were not included because they are outside the scope of this paper. Simultaneously, permanent car-free interventions were also not included, as these are a different topic of study altogether, and were recently reviewed elsewhere [55].

It is essential to distinguish between observed health pathways, e.g., reduced air pollutants that could lead to reduced morbidity and mortality according to the literature, and actual health impacts, e.g., the observation of reduced morbidity and mortality. Given the short-term nature of car-free days and events, it is questionable whether the health effects are large enough, or had enough time to manifest, to be observable using scientific methods, as the health pathways leading to changes in the health effects may return to their baseline for the rest of the year. For this reason, there could be a research bias in the study of health pathways over health effects, with the latter being less studied.

## Results

### Literature overview

Overall, 43 studies and articles were reviewed. Of the literature reviewed, 23 discuss changes in one or more health pathways while two reported on health outcomes. Fifteen articles came from peer-reviewed journals, while the remaining eight were pulled from gray literature (including conference papers). Ten peer-reviewed articles focused on air pollution, three focused on physical activity, and one focused on noise pollution and social inclusion, respectively. Of the gray literature, six discussed air pollution, one discussed both noise and air pollution, and another one discussed social inclusion.

Of the 16 articles that measured or remarked on air quality changes of car-free initiatives, the results ranged from worsened air quality to substantial air pollution reductions. Each of the remaining seven studies and articles concluded positive health pathways including higher rates of physical activity and lower levels of sedentary behavior compared to non-participants, reduced noise levels at the street-scale (less noticeable at larger scales), and other positive impacts among participants such as social interaction, finding entertainment, and supporting community cohesion. These articles and studies are further detailed in Table 2.

**Table 2:** Health Pathway Impacts Identified in Peer-Reviewed Articles and Gray Literature

Case Location	Type of Article	Frequency of Car-free activity	Motivation	Scale	Health Pathway	Impact	Comparator	Reference
Milan, Italy	Peer-reviewed journal article	Single car-free event	Measure impact of transportation on air pollution	City center closed to vehicles	Air pollution	Reduction in CO (35%), NO_2_ (35%), and PM_10_ (13%)	Pollution concentration night before car-free day	Vecchi et al. (2007) [56]
Turin, Italy	Peer-reviewed journal article	Weekly car-free event	Reduce air pollution	City center closed to vehicles	Air pollution	No change in PM concentrations	Daily PM concentrations during a 7-week period	Casale et al. (2009) [57]
Bogotá, Colombia	Peer-reviewed journal article	Weekly *Ciclovía* event	Provide opportunity for physical activity and active transportation	70 miles of street closure in city	Physical activity	*Ciclovía* provides opportunity to attain recommended levels of physical activity	N/A	Sarmiento et al. 2010) [58]
Medellin, Colombia	Peer-reviewed journal article	Once annually	Reduce air and noise pollution and provide opportunity for physical activity	Several main streets through city	Noise pollution	Slight reductions of ambient noise levels ranging from 0.1 to 1.2 dBA	Median ambient noise levels on non-car-free day	Rendon et al. (2010) [59]
Brussels, Belgium	Peer-reviewed journal article	Once annually; multiple observations at multiple sites	Reduce air pollution	Restricted use of all private vehicles in the city.	Air pollution	PM concentrations on car-free days were 3x higher	Mean PM concentrations on normal Sundays and weekdays	Vanderstraeten et al. 2010 [60]
Brussels, Belgium	Peer-reviewed journal article	Once annually	Reduce air pollution	Restricted use of all private vehicles in the city.	Air pollution	Reduction in NO (95%), black carbon (80%) and increase in PM_10_ (150%)	Mean PM concentrations on normal Sundays and weekdays	Vanderstraeten et al. (2011) [61]
Hangzhou, China	Peer-reviewed journal article	Once annually	Reduce air pollution	Unknown	Air pollution	Reduction in CO (20.60%), NO (23.3), NO_2_ (18%), PM_2.5_ (32.6%)	Non-car-free day	Xu et al. (2013) [62]
Hong Kong	News Article	Inconsistent	Street closure due to street protests	Unknown	Air pollution	Reduction in NO (44-52%)	Pollution concentrations days before protest	South China Morning Post (2014) [63]
Po valley, Italy	Peer-reviewed journal article	Inconsistent	Traffic restrictions due to unhealthy air quality	City center closed to vehicles	Air pollution	No change in CO, NO, NO_2_	Pollution concentrations on Non-Car-Free day	Masiol et al. (2014) [46]
Gurgaon, India	News Article	Weekly car-free event	Encourage active transportation	No parking on four main streets	Air pollution	Reduction in PM_2.5_ (21%)	Unknown	Kholi (2015) [64]
Hong Kong	Peer-reviewed journal article	Inconsistent	Street closure due to street protests	Unknown	Air pollution	Reduction in NO (∼80%), NO_2_ (50%), PM_2.5_ (difference unclear), and PM_10_ (difference unclear)	Daily mean air pollution concentrations before and after the protests	Brimblecombe & Ning (2015) [65]
Los Angeles, USA	Online Article	Annual car-free day	Reduce air pollution and provide opportunity for physical activity	Unknown	Air pollution	Reduction in PM_2.5_ (49%) and UFP (21%)	Air pollution concentrations on a typical Sunday	UCLA (2015) [66]
Paris, France	News Article	Single car-free event	Reduce air pollution	~30% of streets closed	Air pollution	Reduction in NO_2_ (20-40%)	Air pollution concentrations on a typical Sunday	Willsher (2015) [67]
Hong Kong	Peer-reviewed journal article	Inconsistent	Street closure due to street protests	Unknown	Air pollution	No change in PM_2.5_ concentrations^***^	Urban background site with normal traffic conditions during the protests	Lu et al. (2016) [68]
Lumajang, Indonesia	Peer-reviewed journal article	Weekly car-free event	Unknown	City center closed to vehicles	Social inclusion	Participants derived pleasure from the social opportunities at car-free events	N/A	Irdiana (2017) [69]
Brownsville, Texas, USA	Peer-reviewed journal article	Four to six times per year	Provide space and opportunities for physical activity	Three miles of street closure through city center	Physical activity	17.3% of attendees met weekly recommended levels of physical activity at the car-free day event	N/A	Salazar-Collier et al. (2018) [70]
Shah Alam, Malaysia	Conference Paper	Unknown	Measure impact of TRAP	Street closure on university campus	Noise and air pollution	Noise reduction of 4.7 (+/-1.74) dBA **and CO_2_ reduction of 13.57 (+/- 4.87 parts per million)	Hourly measurements on a non-car-free day	Gharsheen et al. (2018) [71]
Bogor City, Indonesia	Peer-reviewed journal article	Weekly car-free event	Reduce air pollution	Single street closure	Air pollution	No substantial change in concentration of CO, NO, SO_2_, PM_2.5_, PM_10_, and ultrafine particulates (UFPs)	Air pollution concentrations on non-car-free days	Rachmawati et al. (2019) [72]
Bogotá, Colombia	Peer-reviewed journal article	Weekly *Ciclovía* event	Provide opportunity for physical activity and active transportation	74.6 miles of street closure in city	Physical activity	Increased moderate-to-vigorous physical activity (+6 minutes) and reduced sedentary behavior (-19 minutes) for children who frequently participate in *Ciclovía*	Children who do not frequently participate in *Ciclovía*	Triana et al. (2019) [73••]
Indonesia (Jakarta, Bandung, Garut, Malang)	Conference Paper	Varies by city	Reduce air pollution, traffic congestion, provide space for physical activity	Unknown	Social inclusion	Participation in car-free initiative was motivated by social opportunities	N/A	Prabowo et al. (2019) [74]
Hong Kong	Peer-reviewed journal article	Inconsistent	Street closure due to street protests	Unknown	Air pollution	No change in CO, PM_2.5_ or PM_10_^***^, reduction in NO_2_ (50% for 4-6 hours during the June 2019 protests)	Air pollution concentrations at background sites during the protests	Brimblecombe (2020) [75]
Makassar, Indonesia	Conference Paper	Unknown	Reduce air pollution	Car-free day on three roads with diverted traffic	Air pollution	No change in CO concentrations	Hourly air pollution concentrations on a non-car-free day	Zakaria et al. (2020) [76]
Kigali, Rwanda	White Paper	Bi-weekly *Ciclovía* event	Reduce air pollution	Major roads closed to motor vehicle traffic	Air pollution	Car-free days resulted in a 15% decrease in PM_2.5_ while local COVID-19 travel restrictions resulted in a 33% decrease in PM_2.5_	Daily mean air pollution concentrations on non-car-free days/ days before lockdown	Kalisa et al. (2021) [77••]

*Statistical significance (at the 90% level)

**Statistical significance (at the 95% level)

***No test for statistical significance

Two studies quantified the health impacts of car-free interventions. The one study that reported on actual health outcomes monitored physical activity levels and obesity indicators of child participants in Bogotá’s *Ciclovía* [73••]*.* Another study estimated the health benefits from reduced levels of air pollution in Kigali, Rwanda during car-free days and COVID-19 lockdowns [77••]. The remaining 21 articles and studies simply quantified the changes in pathways to health: air or noise pollution levels, time spent physically active, or self-reported feelings of social inclusion. While the health effects of exposure to air and noise pollution, sedentary behavior, and social isolation are relatively well understood, connecting these health pathways to measurable health outcomes by conducting longitudinal studies has not been done in the context of car-free initiatives and would be an improvement for future studies.

### Documented effects on pathways to health and health effects

From Table 2, it is apparent that ambient concentrations of nitrogen oxide (NO) and black carbon (two pollutants commonly associated with road transport) are reduced up to 95% and 80%, respectively, during car-free periods. Meanwhile, reductions of carbon monoxide (CO), CO_2_, and nitrogen dioxide (NO_2_) were smaller, reaching up to 60% in some cases. Some studies showed that the car-free day or event did not reduce pollution concentrations compared to days without car-free programming, or that the reductions were not statistically significant, in which case “no change” is reported, rather than the number itself [46, 72, 76]. Three more studies reported “no change,” but did not test for statistical significance, in which case it is noted that statistical significance was not discussed [57, 68, 75]. Simply comparing the percentages in the table is insufficient, however, as the different contexts, measurement locations, comparators, and study procedures result in different outcomes in addition to the differences in the car-free days and events themselves. Future studies could improve the ability to compare the impact of car-free initiatives if they made appropriate adjustments in their analysis to account for these contextual factors and other confounders.

The largest reductions, as reported by Vanderstraeten et al. (2011), were measured using ambient pollution concentrations from annual car-free days in Brussels from 2002 to 2009. The annual car-free day in Brussels includes the entire metropolitan region and lasts from 9:30 am to 7 pm. Only certain vehicles (taxis, emergency vehicles, disabled, diplomates, etc.) are allowed to drive, at a maximum speed of 30 km/h. Measurement sites were located in a traffic tunnel and at a roadway environment where concentrations are generally very high, and traffic is the primary source of emissions.

The precarity of mitigating high levels of air pollution is exemplified in Vanderstraeten et al. (2010) which reported that PM concentrations increased in Brussels on several car-free days, despite the initiative covering the whole metropolitan area. The authors argue that the increase in total PM concentrations was in part caused by a change in wind direction, which may have blown air pollution and secondary aerosols from the western part of Belgium toward Brussels. Interestingly, one of the most harmful PM fractions, black carbon, approximately halves on the car-free day despite a substantial rise in PM levels. The authors concluded that black carbon is a more accurate way of measuring particulate matter pollution produced by traffic than total PM concentrations, which can come from a wide variety of sources [78].

Kalisa et al. (2021) analyzed PM_2.5_ concentrations datasets at three sites in Kigali on car-free Sundays in Kigali, Rwanda to determine the impact of car-free days and COVID-19 lockdown travel restrictions. The Rwandan government expects urbanizations rates to increase from 18% to 70% by 2050 which may induce automobile travel demand [79]. To avoid worsening air quality, policies focused on reducing automobile travel have gained interest. In 2016, a car-free day was established in Kigali every second Sunday, closing major roads in the city to automobile traffic.

Car-free Sundays produced mean PM_2.5_ reductions of 15% and overall traffic activity decreased by 27% compared to normal traffic Sundays [77••]. A natural experiment to measure the impact of motorized traffic on air pollution was possible due to COVID-19 lockdown travel restrictions during March–June 2020. Travel restrictions were implemented March–May and followed by looser restrictions from May–June 2020. During the first lockdown, travel activity decreased by 80% and PM_2.5_ concentrations decreased by 33% [77••] compared to median PM_2.5_ concentrations from January–February 2020. The second lockdown, which permitted delivery vehicles, essential services, and government vehicles to travel, decreased travel activity by 40% and PM_2.5_ concentrations were reduced by 21% [77••]. The authors estimated the public health impact of car-free Sundays was 1000 saved disability-adjusted life years (years of healthy life lost due to disability) and 600 avoided hospital admissions from 2021 to 2025 [77••].

Sarmiento et al. (2010) argue *Ciclovía* is a substantial and important opportunity for physical activity population-wide. *Ciclovía* is a car-free event that promotes cycling by closing streets to motorized traffic. Every Sunday from 7 am to 2 pm, about 121 kilometers of roads are closed to motorized traffic as 600,000 to 1.4 million people participate in *Ciclovía* [80]. While *Ciclov*í*a* originated in Bogotá, Colombia, it has become a best-practice adopted in cities around the world [81]. In Bogotá, Sarmiento et al. (2010) estimated that 13% of population-wide weekly physical activity was attained during *Ciclov*í*a*. This estimate was derived from an equation (Equation 1) measuring the share of population-wide total physical activity attained from participation in *Ciclov*í*a*. The average attendance at *Ciclovía* is one million people who, based on a survey of participants, engage in roughly 140 minutes of physical activity during *Ciclov*í*a.* If the entire population of Bogotá (6.8 million) engaged in the weekly recommended total 150 minutes of physical activity, then *Ciclov*í*a* produces 13% of weekly minutes spent engaged in physical activity*.* A similar calculation of physical activity attained during *Ciclov*í*a* in Brownsville, Texas reported that 17% of participants satisfy weekly physical activity recommendations through *Ciclovia* [70].

Equation 1. Estimate of population-wide weekly physical activity attained during *Ciclov*í*a*

1 $$\frac{\mathrm{1,000,000}\times 140}{\mathrm{6,840,116}\times 150}=0.1364=13.64\%$$

The assumption that each citizen of Bogotá attains 150 minutes of physical activity (used in Equation 1) is unfounded, however, thus it can be inferred that the share of population-wide physical activity represented by *Ciclov*í*a* may be higher than 13%. Furthermore, the official recommended level of physical activity is 150 minutes of moderate-intensity activity (or 75 minutes of vigorous-intensity activity), preferably spread throughout the week [82]. While *Ciclov*í*a* may generate the opportunity to engage in physical activity for an extended period, the once per week occurrence means that the event does not support opportunities for physical activity throughout the week as the physical activity guidelines prefer.

Despite increased levels of physical activity among *Ciclov*í*a* participants compared to non-participants on Sundays, obesity rates and body mass index scores are higher among child *Ciclov*í*a* participants in Bogotá than child non-participants [58]. Children participating in *Ciclovía* were more likely to satisfy physical activity guidelines on Sundays than children who did not frequently participate, although differences in physical activity levels between the study and control group were trivial during the week [73••]. Overall, children who participated in *Ciclovía* were engaged in 6 more minutes of moderate-to-vigorous physical activity and 19 less minutes of sedentary behavior than their non-participant counterparts on Sundays [73••]. Although frequent *Ciclovia* participants were substantially more active than non-frequent participants, no beneficial impact was reported on weight among participants. In fact, children frequently participating in *Ciclovía* were more likely to be overweight than children who participated sporadically or not at all (28.3% vs 20.4%). These results were concluded after administering a questionnaire to study participants (children and their parents) to gather data on demographics, family health, diet, and lifestyle characteristics. This may result because *Ciclovia* occurs once per week and may not offset unhealthy behaviors (physical activity, diet, and sedentary time) during the rest of the week [73••] . 

The impact of car-free days on noise levels is variable between studies, largely due to the scale of observation for each study. Gharsheen et al. (2018) measured noise levels at the building scale, placing monitors in the foyer of two buildings, one within and one outside the car-free zone. While noise levels inside the building within the car-free zone were measured to be reduced by 4.7dBA compared to non-car-free day noise levels (7.2% of non-car-free day mean noise level), noise levels inside the building outside the car-free zone were increased by 1.7dBA compared to non-car-free day noise levels, likely due to displaced traffic. Rendon et al. (2010) recorded noise levels on four streets during a car-free day and observed slight reductions of ambient noise levels ranging from 0.1 to 1.2 dBA, compared to non-car-free day noise levels.

Social inclusion was the focus of only two sources reviewed in this paper, both coming from car-free events in Indonesia. Both studies surveyed participants at car-free events about their motivations to attend and the perceived benefits of attending. In each case, survey respondents were motivated to participate because they were seeking social interaction or attracted by the programs and activities associated with the car-free event (food, shops, and places to play) [69, 74].

Several car-free events we reviewed in the literature were not planned as such. For example, protests in Hong Kong resulted in an interesting range of studies by Brimblecombe (2020), Brimblecombe and Ning (2015), and Lu et al. (2016), while in London, streets were closed for its famous marathon and NO_X_ pollution levels decreased by 89% compared to non-marathon days [83]. During protests in Hong Kong that shut down car traffic on certain streets, NO and NO_2_ concentrations were reduced by as much as 80% and 50%, respectively [65, 68]. Such opportunities provide unique case studies to measure the effects of temporary traffic bans and shed light on the potential impacts of car-free initiatives.

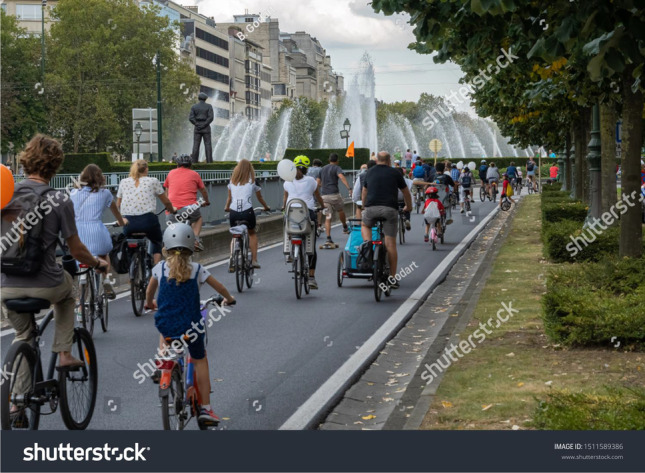



Brussels, Belgium—September 22, 2019: Cyclists in the streets of Brussels during the “Journée sans voiture en Belgique” (Bruxelles), source: https://www.shutterstock.com/image-photo/brussels-belgium-september-22-2019-cyclists-1511589386

**Table:** 

Bogotá, Columbia has hosted an official car-free event known as *Ciclovía* each Sunday since 1982 [84]. *Ciclovía* began as an “urban experiment” in 1974 to demonstrate not only the utility of cycling for transportation, but also the relationship between environmentalism and urbanization in Bogotá [81]. The institutionalization of *Ciclovía* was meant to maintain a culture of cycling behavior and support public health [81]. Every Sunday from 7 am to 2 pm, about 121 kilometers of roads are closed to motorized traffic as 600,000 to 1.4 million people participate in *Ciclovía* [80].	

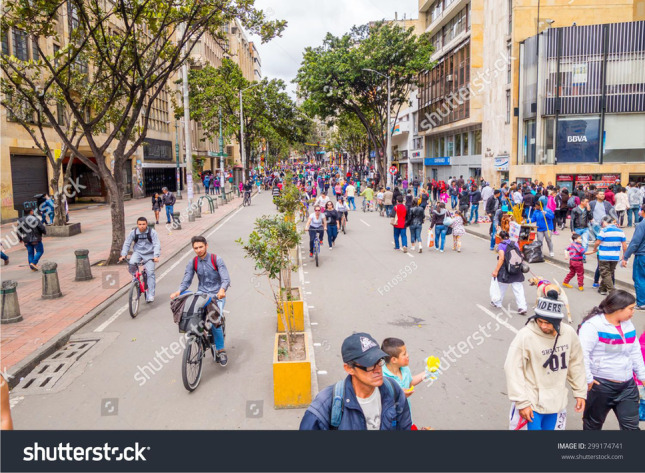



BOGOTA, COLOMBIA—FEBRUARY 9, 2015: Unidentified Hispanic pedestrians and cyclists moving through city street Candelaria area Bogota, source: https://www.shutterstock.com/image-photo/bogota-colombia-february-9-2015-unidentified-299174741

## The characteristics of a successful car-free day

We think a successful car-free day or event is one that restricts motor vehicle travel, without simply displacing it to another neighborhood and promotes active transportation instead, therefore increasing physical activity and improving other pathways to health [22••]. It is important that car-free days or events do not displace traffic and transportation-related air and noise pollution, but holistically mitigate the environmental stressors and health burdens of motorized transport in urban areas. Success can thus be measured in terms of improved pathways to health such as reduced air pollution and reduced noise and improved physical activity and social interactions in addition to improved health outcomes where possible to measure [22••]. Pathways to health should be considered holistically and not in isolation as the best outcome would be improvements in all, or at least net improvements even if there were some losses. Also, importantly, losses should not be exacerbated in vulnerable populations, for example by displacing traffic to low-income and ethnic minority communities who are already burdened by and susceptible to environmental stressors.

In at least 6 cases in the reviewed literature [46, 71, 72, 74, 76, 77, 85], issues with diverted traffic were reported. Perhaps unsurprisingly, none of these studies found significant noise or air pollution concentration reductions across the board, with some studies even finding increases. For example, in Johor Bahru, Malaysia, CO_2_ reductions at one site within a car-free zone were more-than offset by CO_2_ increases in another site outside the car-free zone; at the same time, while some noise reductions occurred at the site within the car-free zone, diverted traffic increased noise levels at the other site outside the car-free zone [71]. Similarly, in Indonesia, some car-free days are treated more as “places for commerce and snacks,” with most people still arriving at the car-free area by motorized transportation [74]. It is thus important for car-free days and events to limit diverted traffic, which can cause disproportionate traffic, congestion, and emission increases in non-car-free areas, which erodes not only the purpose but can also erode the support for such events. Traffic diversions may also exacerbate environmental injustice, if the diverted traffic goes to neighborhoods of ethnic minorities and lower socioeconomic status populations.

Some of the most successful car-free days and events in terms of health pathways seem to be in Brussels and Hong Kong, which happen to be large in scale: the former covers the whole metropolitan area, while the latter went on for weeks and closed off many busy streets. In all likelihood, the effects of car-free days and events in larger areas are thereby not only stronger but their positive pathways affect a larger population and potentially reduce the likelihood for environmental injustice and health inequity throughout an urban area. For successful car-free days in terms of health pathways, it is thus crucial to make sure that total motorized traffic is reduced by designating a large area as car-free and supporting alternative travel modes. 

Public activism was a factor in establishing Bogotá’s *Ciclovía*, with a local cycle group initially campaigning for a localized street closure for one day [81]. Sarmiento et al. (2010) noted that public pressure also played an important role in influencing the development of the *Ciclovía* when changes to the initiative were proposed. A key factor to the longevity of Bogotá’s *Ciclovía* is the sustained political support which has persisted since the 1970s, with the scheme expanding in size since its inception [58].

## Facilitators and barriers for car-free day implementation

Wider political and social factors need to be considered for the successful implementation and longevity of car-free days and events. A number of facilitators and barriers to the successful implementation of car-free days have been identified in the literature.

First and foremost, the successful creation and implementation of car-free days are heavily dependent on political will. Loo (2017) notes that no national legislation exists which requires municipalities to implement car-free days, and therefore any action depends on political support at a municipal level. Municipalities are the principal actor in organizing car-free days, and thus commitment at the local political level is needed to commence the initiative and ensure its success [58, 81, 84]. Evidence from *Ciclovía* initiatives in South America has shown that a decline in political support can lead to the initiatives becoming inactive or delayed, ultimately reducing success and impact of the initiatives [58]. Financing is a key element in demonstrating political support and a lack of financial backing can be a key barrier, particularly in high-income countries where costs tend to be higher (e.g., policing costs) [58].

The manner in which car-free days are managed can also be an important factor in facilitating long-term success. Coordination between different departments within municipalities (e.g., transportation, policing, engagement) appears vital in facilitating effective implementation [58, 81]. Indeed, in their examination of C*iclovía* schemes*,* Sarmiento et al. (2010) noted that intersectoral collaboration between municipal departments and non-government agencies is a key determinant of the development and continuity of many C*iclovía* schemes. Similarly, strong collaboration between the public and private sector is important for delivering successful and attractive car-free days [58]. Creating strong communications and marketing for car-free days is also noted as a facilitator for a successful event, helping to generate interest and gain support [58, 86]. For example, in the case of Madang, Indonesia, Hussein (2016) demonstrated that building a positive image of a car-free day can help to encourage people to return to future events. 

Public opinion and involvement in the planning of car-free initiatives can be a key factor for successful implementation. In some cases, citizen groups are driving the creation of car-free days, such as Bogotá [81] and Paris [87]. C*iclovía* in Bogotá was spawned by the citizen group Pro-Cicla who was advocating for cycling as a viable commute mode, while the car-free day in Paris was originally proposed to Mayor Anne Hidalgo in a letter written by a citizen group. Community support has been shown to be important in ensuring the continuation of car-free days [58]. Even in cases where political will for car-free days is lacking, support from local citizens can lead to temporary street closures [88]. This was the case in Toronto, where grassroots initiatives achieved localized street closures despite a lack of support from the municipality for a larger-scale car-free day [88]**.**

Barriers which limit citizen participation can lead to unsuccessful or unsustainable car-free days and other car-free initiatives. Concerns over personal safety in car-free schemes—particularly for women—can limit participation and result in opposition [80, 89, 90]. However, this may be limited to specific contexts, with concerns about the safety of car-free days most frequently documented in Southeast Asia [89, 90]. Sarmiento et al. (2010) identified that both real and perceived lack of access to car-free areas has been a barrier to public participation in certain C*iclovía* schemes, limiting the potential public health benefits from these events.

The view of businesses and interest groups can play an important role in facilitating or hindering action on car-free initiatives [91–93]. Sarmiento et al. (2010) noted how private sector stakeholders can play an important role in providing financial support, as was the case in Bogotá, Columbia; Brasilia, Brazil, and Santiago, Chile**.** Businesses have been found to commonly object car-free schemes, however. In Kraków, Poland, and Oslo, Norway, businesses were opposed to new car-free areas being established in the city center [92–95]. Indeed, such opposition from businesses (including the car manufacturing lobby) has been shown to result in the reversal of measures to reduce car use [96, 97]. While businesses can indeed be a barrier to car-free measures, McKibbin (2014) notes that businesses can be supportive of car-free days, potentially due to the increased economic activity often seen during car-free days [84, 92]. What appears important in gaining the support of businesses is involving and engaging business stakeholders in the process of developing a car-free scheme, focusing on issues such as consultation, clear communication, and willingness to make changes based on feedback [88, 92]. For example, Even-Har and Hostovsky (2006) highlight the role of consultation and engagement with businesses and other stakeholders as an important factor for addressing concerns regarding Montréal’s car-free day event [88].

## Barriers not acknowledged in academic literature

Having reviewed literature on barriers to car-free days and events and similar car-free initiatives, it is important to note that certain barriers received less attention in academic literature. We turned to the broader gray literature to consider how barriers to car-free planning compared to barriers to car-free days and events. In particular, the influence of community opposition to car-free and low-traffic initiatives is routinely noted in the gray literature and appears to be a key factor in the failure of such schemes. A recent example comes from New Orleans, where French Quarter residents rallied against proposals to turn the historic neighborhood into a pedestrian area. All around the quarter, residents posted yard signs calling to “save their neighborhoods” and oppose the permanent exclusion of cars from certain streets (referred to in the city as pedestrian malls, see figure below). Residents cited a number of reasons for the opposition including that pedestrian malls in the French Quarter would cater more for tourists than residents, and would reduce or eliminate the residential function of the quarter, in addition to making it more difficult for lower socioeconomic status workers to get to and leave the quarter, as they could not afford living there and need to commute to their jobs [98]. Concerns about traffic diversion to other neighborhoods in New Orleans have also been voiced and are in line with the literature reviewed above.

Despite evidence that schemes to reduce the use of cars receive public support [99], those who are opposed can have a large impact on the success of such schemes. Direct action from those opposed can result in the delay or even reversal of such schemes [98, 100, 101]. A claim made by those opposed is that schemes to limit car use disproportionately benefit higher-income groups, leads to gentrification, and can cause issues for low-income areas, such as re-routing of traffic into those areas [101] and low-income groups not being able to access job opportunities. This raises questions over differing levels of power in consultation processes and whether all groups in lower-income areas are receiving due consideration and attention in transportation planning. As such, greater consideration may be needed to ensure the needs of all groups are accounted for in decision-making processes [102]. Despite less evidence of community opposition to car-free days than other car-free initiatives, the experience from other initiatives may serve as a useful learning exercise to consider for the planning and implementation of car-free days and events.

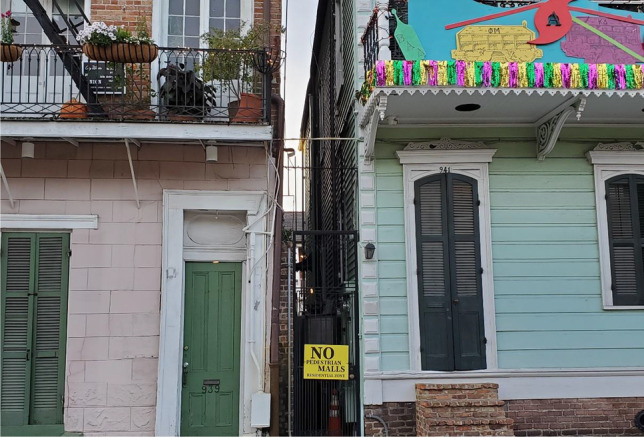



New Orleans “No Pedestrian Malls” signs in residential areas of the French Quarter, February 2021, source: own photograph

## Discussion

Car-free days and events are present in many countries around the world including Colombia, China, Belgium, France, Hong Kong, Indonesia, India, Italy, Malaysia, and the USA. In some places, these initiatives have been established for several decades. Although the scale and scope of these events may vary by location, the beneficial impacts of car-free initiatives are becoming better understood and their popularity appears to be increasing. The recent COVID-19 pandemic also highlighted a renewed appeal for less motor vehicle traffic in cities, increased walking and biking and the reallocation of space for the public including outdoor café and restaurant seating and entertainment opportunities. A review of the literature produced a geographically diverse documentation of car-free initiatives. Most common were academic papers discussing car-free days and events in Europe, Southeast Asia, and South America. Gray literature focused more on car-free days and events in North America, but these were more limited. This does not mean, however, that the majority of car-free days and events are held in these areas, as there might be more research in high- and middle-income countries compared to lower-income countries, where events might be held but not documented. The literature that was reviewed evaluated the impact of car-free initiatives along four pathways to health: air pollution, noise pollution, physical activity, and social inclusion. Other relevant pathways including, utilization of blue, green, and public space, urban heat, road crashes and injuries and stress [22••] were not evaluated in the included studies, but it is important to note that our literature search was non-systematic and as such, we might have overlooked some of those reports.

The initial stages of the formation of the car-free initiative ultimately determine the scale of car-free initiative. In this paper, scale refers to the frequency, duration, and geographic size at which the car-free initiative is implemented. The impacts reported in reviewed literature suggested an influence of the scale of the car-free initiatives, but reported impacts also vary based on the method of measurement applied in each study, in addition to the metrics selected. Air pollution, for example, may have been reduced along restricted routes where the car-free initiative was implemented, but may have increased on adjacent roads as some of the traffic was re-routed, failing to reduce emissions and air pollutant concentrations. A more comprehensive—and successful—approach to organizing a car-free day targeted at reducing air pollution may rely on strategies that aim to reduce the use of cars while expanding opportunities for alternative travel modes to effectuate mode shifts, based on the input and preferences of communities most affected by adverse air pollution exposures and by the proposed changes. Indeed, there is evidence to suggest that transport policy packages with multiple complementary measures are more effective in achieving goals than isolated policy measures [103, 104]. In some instances, such as in Milan, Italy, and Brussels, Belgium, the impact on air pollution was negligible when measuring levels of PM (10 and 2.5). In both cases, PM concentrations were almost equal inside and outside restricted travel areas [61, 105]. However, reductions of up to 78% were measured for concentrations of black carbon, prompting conclusions from both studies that PM may not be the most suitable indicator of TRAP mitigation as a result or the car-free initiatives.

Similarly, physical activity was a common motivation for car-free initiatives, however, its health impacts were difficult to measure, perhaps given the low frequency of car-free initiatives [73••]. Conclusions may also be affected by the metric of physical activity selected. Although several studies did find participants in car-free initiatives attain high levels of physical activity, the most frequent initiatives occurred once per week, and only for a portion of the day, which limits the impact on a person’s weekly physical activity rates. Longer and more frequent car-free initiatives may improve the ability to measure physical activity health impacts. Also, while minutes of physical activity might differ between participants and non-participants on the car-free days, on normal days, differences seem to disappear which might explain the lack of differences in sedentary time and body mass index reported by some studies [73••].

The formation of car-free days and events varied between cases and are influenced by several factors. Political will, specifically at the municipal level, is crucial to adopting car-free initiatives. The political will for car-free initiatives is often influenced by specific motivations such as improving air quality, providing opportunities for physical activity, reducing traffic congestion, or cutting down on greenhouse gas emissions and mitigating climate change [97]. Political will is substantially hindered if there is a lack of coordination between municipal departments and agencies (transportation, police, etc.), which we have identified as a potential barrier to the implementation of car-free initiatives.

A car-free initiative, and the goals and purpose of the initiative, must also be marketed effectively to engender the support and participation of the public. In several cases, however, car-free initiatives are prompted by organized public efforts, which may supplement the initial public will necessary for the adoption of such policies. Local business opinions also influence the formation and sustainability of car-free initiatives.

Access—both to the car-free event and to destinations within a car-free city area—is central to the formation and adoption of car-free initiatives. One paper that discussed barriers and facilitators to car-free initiatives recognized several factors related to accessibility that are essential for car-free policy implementation, including: a paradigm shift in transportation planning to prioritize accessibility over mobility, providing convenient transportation alternatives to maintain access, and securing support from stakeholders by ensuring an acceptable level of access is maintained [97].

Other important considerations include gentrification, environmental justice, and health equity issues, which can be improved or worsened by the car-free initiatives. Resistance to sustainable development agendas globally highlights the concern from communities that the impact of well-intentioned policies could be personally harmful [106]. Governments and corporations have identified an advantage in pursuing urban sustainability projects that produce spaces of consumption, tourism, and commerce which can threaten existing communities with displacement and changing the fabrics of their neighborhoods [106]. The proposal of car-free initiatives may raise similar concerns.

While the result of car-free initiatives may be a temporarily car-free environment, the ideal of car-free space is not mentioned as a top priority among the reviewed literature. The car-free initiatives referenced in the literature most often reflect a policy preference for reducing environmental or health-related burdens, as opposed to reducing the use of cars for travel. Gössling (2019) advises against taking a stance against cars, as such arguments may be interpreted as antagonizing, and that stance is generally highly controversial. Instead, positive messaging about the purpose of car-free initiatives—such as health benefits—may be more palatable and convincing to a broader audience [21••]. 

In particular, we may have a unique opportunity to seize car-free travel in a post coronavirus world. Transportation patterns have been disrupted and communities have been exposed to new mobility patterns, which may be sustained if they are catered to. For example, some reports suggested that strategic designs, such as expanding sidewalks, redesigning transit, widening and cordoning off bike lanes, installing street furniture and public art, and increasing vegetation, can help car-free cities gain and sustain popularity as we emerge from the pandemic [107, 108].

With the exception of Triana et al. (2019) and Kalisa et al. (2021), the existing literature did not measure the health impacts of car-free initiatives [73••]. One possible explanation is that the health impacts of, say, reduced air and noise pollution or increased physical activity, are already well documented in other literature. Furthermore, spontaneous car-free events, such as protests, do not allow research *ex ante*. Additionally, and perhaps most importantly, none of the programmatic car-free initiatives occurred more frequently than once per week, making measurement of health impacts difficult and limiting their effects on a year-round basis. The inability to measure health impacts suggests the utility of existing car-free initiatives lies primarily in showing the public alternative urban experiences that exclude cars and promote active transportation and reallocation of urban space. This way, the primary goal of car-free days and events is to spur changes beyond the day itself, by inspiring change toward healthy transportation and to gather support for permanent car-free urban environments. Future longitudinal studies can, however, attempt to measure health effects of car-free initiatives, considering that they are substantial and frequent enough and account and adjust for a comprehensive list of relevant confounders and contextual variables.

To deliver long-term health benefits, it is important that municipalities implement car-free days alongside wider transportation strategies to prioritize public transportation, walking, and cycling, to facilitate the use of healthier modes of transportation [109]. However, no study measured or projected the impact of car-free initiatives on travel behavior in the long-term, or the influence of car-free initiatives on the adoption of additional car-free policies; an area that remains open for future research.

Overall, while more car-free initiatives are being adopted and the idea of car-free environments may become normalized, a successful car-free initiative is a very complex challenge. Most events require the involvement of many stakeholders which may require compromise and present unforeseen barriers to adoption. Further, the advantage of a car-free initiative must be clearly communicated and achieved. As previously noted, unsuccessful car-free initiatives, and equity and gentrification concerns, may erode the support for continuation of such initiatives. Lastly, safety and accessibility (to destinations and the event itself) determine people’s participation and experience and may influence their support of the car-free initiative [63, 72, 88].

## Summary and Conclusions

In this paper, we reviewed studies which investigated the potential impacts of car-free days and events on human health, either through changes in relevant pathways, such as air and noise pollution, or actual changes in health outcomes, such as body mass index. Car-free days and events are often designed to alleviate the adverse impacts of transportation on the environment and human health, such as the effects of air pollution and noise, or other detrimental externalities of private motor vehicle travel, such as traffic congestion. We found that while many studies investigated changes in air and noise pollution, physical activity, and social inclusion, other relevant pathways were absent from the literature. Furthermore, only two studies actually measured health-related outcomes; the former measuring body mass index and the later estimating reductions in disability-adjusted life years and hospital admissions. We found that the impacts of car-free days and events were highly variable and may be dependent on the scale and goals of each car-free initiative. In some cases, car-free days and events were successful in reducing the concentration of certain air pollutants but had little or adverse impacts on the concentration of others. Issues of traffic diversions, environmental injustice, erosion of residential areas and neighborhood feel, and gentrification were often mentioned in the literature. While car-free days and events can be an attractive, and in our opinion a progressive policy option, the formation and sustainability of such initiatives are highly complex and need to be better understood. Future research could be most useful if it focused on measuring health outcomes associated with car-free days and events through longitudinal studies with appropriate adjustments for confounders and contextual variables. 
